# Exploring the role of task performance and learning style on prefrontal hemodynamics during a working memory task

**DOI:** 10.1371/journal.pone.0198257

**Published:** 2018-06-05

**Authors:** Afrouz A. Anderson, Kian Parsa, Sydney Geiger, Rachel Zaragoza, Riley Kermanian, Helga Miguel, Hadis Dashtestani, Fatima A. Chowdhry, Elizabeth Smith, Siamak Aram, Amir H. Gandjbakhche

**Affiliations:** 1 National Institute of Child Health and Human Development, National Institutes of Health, Bethesda, MD, United States of America; 2 St. Olaf College, Northfield, MN, United States of America; 3 Analytics Department, Harrisburg University of Science and Technology, Harrisburg, PA, United States of America; Technion Israel Institute of Technology, ISRAEL

## Abstract

Existing literature outlines the quality and location of activation in the prefrontal cortex (PFC) during working memory (WM) tasks. However, the effects of individual differences on the underlying neural process of WM tasks are still unclear. In this functional near infrared spectroscopy study, we administered a visual and auditory n-back task to examine activation in the PFC while considering the influences of task performance, and preferred learning strategy (VARK score). While controlling for age, results indicated that high performance (HP) subjects (accuracy > 90%) showed task dependent lower activation compared to normal performance subjects in PFC region Specifically HP groups showed lower activation in left dorsolateral PFC (DLPFC) region during performance of auditory task whereas during visual task they showed lower activation in the right DLPFC. After accounting for learning style, we found a correlation between visual and aural VARK score and level of activation in the PFC. Subjects with higher visual VARK scores displayed lower activation during auditory task in left DLPFC, while those with higher visual scores exhibited higher activation during visual task in bilateral DLPFC. During performance of auditory task, HP subjects had higher visual VARK scores compared to NP subjects indicating an effect of learning style on the task performance and activation. The results of this study show that learning style and task performance can influence PFC activation, with applications toward neurological implications of learning style and populations with deficits in auditory or visual processing.

## Introduction

Working memory (WM) tasks are a set of well-known and well-established cognitive tasks that assess the temporary storage and manipulation of information in the brain in conjunction with neuroimaging, the N-back task has been instrumental in elucidating the relationship between working memory and activation of different brain regions. [1–3]. In the working memory paradigm, the prefrontal cortex plays a significant role in both auditory and visual processing [4–6]. Specifically, tasks related to working memory have been known to activate the left cortical regions [7,8]. Processing of auditory working memory has been shown to be specific to temporal regions BA (47) and the left prefrontal cortex (LPFC) [9], whereas the right prefrontal cortex is involved with non-verbal (visual letter stimuli) processing during the n-back task. Despite its extensive use for the identification of factors which influence both degree and location of neural activation, little research has compared activation patterns by stimulus type, specifically auditory vs. visual. Though it is possible to postulate differences in activation between auditory and visual processing through the comparison of separate studies, it is more accurate to compare instances when both types of stimuli are presented to the same set of subjects.

Previous studies have indicated the region-task specify of the PFC: left prefrontal cortex has a role in encoding during working memory, whereas the right side is involved with memory retrieval [10]. The theory was well defined by Habib et al. [11] as the HERA model, or Hemispheric Encoding/Retrieval Asymmetry. Based on HERA model, during the working memory task, the type of the stimuli (auditory vs. visual) can affect the activation in prefrontal cortex. It should be noted that differences in information processing during a working memory task can be dependent on an individual’s encoding and learning strategy. Based on the multisensory representation of working memory, there are multiple ways to encode the stimuli presented during a task (i.e., audio, visual, or audiovisual) [12]. Delugo et al. [13] suggested that during both verbal and non-verbal presentation conditions, the actual content of the stimulus is encoded in a form of verbal information. For example, a person might see the shape of a square, but encode what they have just seen as the word “square”. They iterated that the format in which information is presented is not necessarily the format in which the information is encoded. This implies that learning style, or an individual’s proclivity toward certain types of stimulus, may play a role in processing different stimuli within the working memory paradigm. Gevins et al. [14] showed that while performing an n-back task subjects with a verbal cognitive style had greater activation in the left parietal region, while subjects with a nonverbal style had greater activation in the right parietal region. Graf et. al. [15] examined the relationship between learning style and cognitive traits such as working memory capacity, where subjects with low working memory capacity had active (i.e. learning by discussing or trying out) and visual learning styles.

Although several studies have included the differences between auditory and visual WM tasks [16–20], very few studies have investigated the role of learning preference and underlying cognitive processing in performance of the WM task. Behavioral studies have shown the link between subjects’ cognitive style and learning preference and their ability to perform visual or auditory tasks [21–23]. For instance, subjects with visual preference can convert the verbal information into that of visual and vice versa. Therefore, how subjects perceive information can ultimately affect the underlying functional regions that is utilized to accomplish the task.

To measure the brain activation in prefrontal cortex, we have used functional near infrared spectroscopy (fNIRS), a neuroimaging modality that has been rapidly increasing in use over the past two decades [24–30]. NIRS uses light in near infrared regions that is sensitive to changes in oxy- and deoxy-hemoglobin, two known hemodynamic factors related to brain function. Therefore, NIRS can be used to assess brain activation in cortical regions and like fMRI, it infers activation from hemodynamic response. Previous studies have indicated a strong correlation between fNIRS and fMRI signals [31–33]. It has several advantages over other imaging modalities such as fMRI or PET which include being non-invasive, relatively inexpensive, and portable. Whereas other imaging methods require subjects to remain completely still, fNIRS has shown greater tolerance for motion artifacts. This allows tasks to be performed without restraints on the subject which may cause excess stress or anxiety. Utility of fNIRS to detect the functional changes in brain during n-back WM task has been shown previously. For instant, Herff et.al [34] have used classification method to quantify the mental work load during the n-back task in PFC. Using fNIRS, activation in PFC has also been shown to increase with the load during n-back task in DLPFC regions and it was possible to detect the functional connectivity during the activation period [35]. Vermeji at al, showed the effect of aging on the time course of the hemodynamic response during performance of verbal n-back WM task [36]. PFC hemodynamic during performance of the n-back task and while walking has also been explored [37], where there was effect of task difficulty in left dlPFC. The findings of this study further suggested that younger and older adults responded similarly to the cognitive load of the n-back task.

The general goal of this study to examine the relationship between the behavioral measures, such as performance and learning style with NIRS measurement in PFC region. This would allow us to see if observed WM activation of the prefrontal cortex using fNIRS are related to how individuals process different stimuli types and subjects’ performance. To test such relationship, we conduct auditory and visual n-back WM task while measuring PFC activation. We then include the individuals’ accuracy score as measure of performance level and VARK scores as indicators of learning preference in our analysis, while controlling for age. Specifically, we examine the differences in PFC activation as measured by NIRS, between high performing and normal performing individuals. We further examine the correlation between learning preference and activation in PFC regions. We then study if the learning style is related to the performance level during by comparing the VARK scores in high and normal performing subjects, during auditory and visual n-back WM task. This study can further elucidate the need for more detail analysis of the WM tasks on basis of individual differences and how learning style can be used to better train individuals with learning problems.

## Material and methods

This study was approved by an Institutional Review Board at the National Institutes of Health.

### Subjects

29 healthy adults with age range of 20–59 years (Female = 15, mean age = 36.2, SD = 12.35) were recruited from the community. All subjects completed a written, informed consent prior to their participation.

### Task

**Fig 1:**
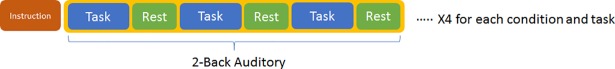
N-back working memory task for auditory and visual task for each 0-back and 2-back conditions. The task blocks were each 34-seconds long with the resting period of 15-seconds in between.

Subjects completed both 0-back and 2-back conditions for both auditory and visual (letter) n-back tasks. The task was performed inside the quite room. Prior to start of the task, adjustment of audio sounds through two speakers was done to ensure that subjects can properly hear the stimuli. Subject were also asked if they could easily see the letters on the screen. Each task contained 3 blocks (total of 18 targets and 33 non-target stimuli for each condition and task) in which subjects would hear the letter from a set of speakers or observe it on a screen. The inter-stimulus duration was 1.5 seconds, while each stimulus was presented for 500 milliseconds. Total of 12 blocks were presented, with the 15 seconds rest periods occurring in between all blocks. Set of 3 blocks, corresponding to specific task and condition were presented together with resting periods in between as shown in Fig 1. The order of the sets was random. The general instruction about n-back task were given to the subjects prior to the placement of the NIRS probe. Instruction for each set was provided before the beginning of each task. Subjects were instructed to click when presented with the target stimulus. Specifically, for the 0-back condition they clicked when the letter B was presented and for the 2-back condition they clicked when the target stimulus letter was the same as the stimulus from two steps before. There was a 15-seconds long resting period between the blocks. The percent correct response and reaction time (in milliseconds) were recorded using the E-Prime 2.0 software package (Psychology Software Tools, Inc).

### VARK questionnaire

Subjects completed the self-assessment learning style VARK (Visual, Aural, Read/Write and Kinesthetic, Version 7.8 (2014), VARK Learn Limited, Christchurch, New Zealand.) questionnaire. The results of this test indicate preferences rather than strengths and can be used for assessing learning styles [38,39]. The higher VARK score is therefore indicative of stronger preference for the given learning style. The questionnaire consists of 16 multiple-choice questions regarding subjects’ preferences when conducting a variety of activities with each choice tending to the certain learning preferences. For instance, subjects will choose how they prefer to learn or do something for the first time or communicate with others. The multiple choice would allow subjects to select the preferred methods to deal with the given situation. The VARK questionnaire and the results focus on how people prefer to receive information or deliver their communication. In this paper we focus on two of the relevant scores: the VARK visual preference score that includes using visual strategies such as those of designs, patterns, and shapes to interpret information and the aural score that is related to preferences for information that is heard or spoken.

## Method

**Fig 2:**
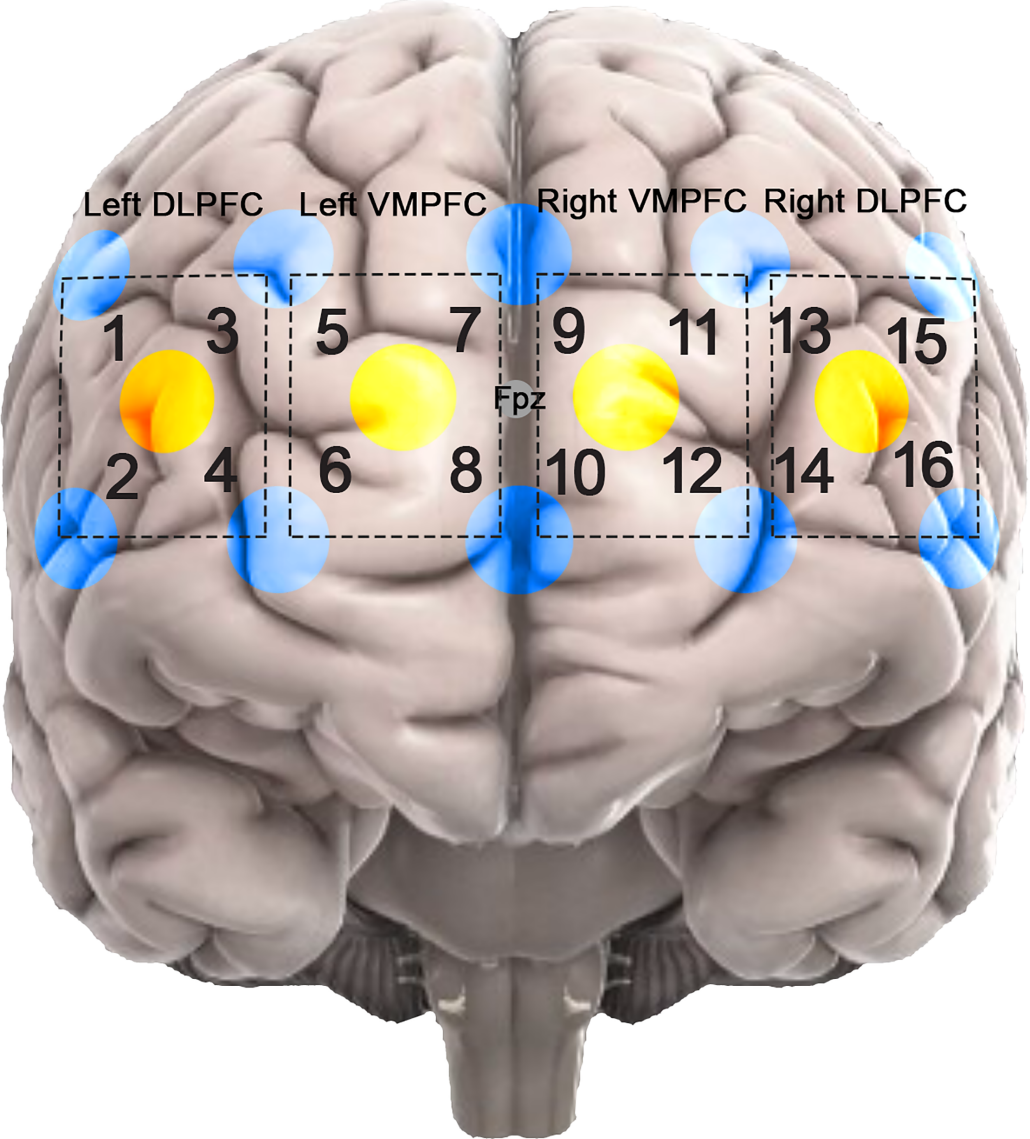
Sensor position on the prefrontal cortex region with respect to Fpz (International 10–20 system). Yellow dots: Sources, Blue dots: Detectors. Numbers indicate the loaction of the channels based on the source-detector pairs. The dotted squares indicate channel parinings.

NIRS data was obtained using a 16-channel NIRS Device (fNIR Devices, LLC) and CobiStudio software [40] to record the raw NIRS signal at wavelengths of 730 nm and 850 nm. The NIRS headband with embedded sensors (4 sources and 8 detectors: 16 channels) was positioned on the subject’s forehead with the center of the band was placed approximately on Fpz location (international 10–20 system) by aligning the center and bottom of the band on the midline and above eyebrow, respectively. The position of the sensor has been used previously in several NIRS studies [40–43].(Fig 2). Channel separation was set at 2.5 cm. The raw NIRS data was preprocessed using fNIRSOFT [44], where Sliding-Window Motion Artifact Rejection (SMAR) algorithm [45] was applied. This algorithm is based on the coefficient of variation method to assess signal quality and is applied to remove the channels or portions of the signal that has been saturated mostly due to motion artifacts or loose channel contact. The method is used for automated data preprocessing and has been used in numerous literature [46–49]. One subject was excluded from study due to motion artifacts that affected the signal during the entire task. The detected signal was converted from raw intensity to change in HbO and HbR using the modified Beer-Lambert Law. As such, we obtain the changes in NIRS signal with respect to the baseline for each participant and constant effects in each participant, such that those by skin and scalp become negligible [28]. In this study we use a source-detector separation that allows for differentiation of signals coming from the cerebrum versus skin. Furthermore, task-related effects on skin blood flow have been shown to be negligible [50–52]. The signals were subsequently detrended for each channel to remove slow changes in the signal and median filtering was applied to remove sharp spikes in the data. We applied a low pass FIR frequency filter (<0.1 Hz) to remove fluctuations in frequencies related to heartbeat and respiration. To avoid edge artifacts, filtered data included samples from 20 seconds prior and post task duration. Both median and low pass filtering has been used previously in literature in pre-processing of the NIRS signal [30,40,44]. We also applied correlation-based signal improvement to remove possible motion artifacts that induce a negative correlation between HbR and HbO signals. HbO signal were used in the analysis as a measure of brain activation since it is associated with hemodynamic response and neural activation. Changes in HbO signal has been shown to be a better correlated of BOLD fMRI signal and have a better signal to noise ratio compared to HbR and has been commonly used in NIRS studies[53–58]. The signals for each condition (0-back and 2-back) and task (auditory and visual) were averaged over their corresponding trials for each channel. For further analysis, and to emphasize on WM demand, signal from 2-back condition trials (involved in WM and attention)were subtracted from 0-back (involved in attention) trials to account for changes related to working memory process, referred to as WM activation, and minimize the activation due to attentional mechanism [59,60]. Upon completion of preprocessing, the sixteen NIRS channels were categorized and averaged by division into four sub-regions: Left dorsolateral/ventrolateral prefrontal cortex (DLPFC) (channels 1–4), Left medial frontopolar prefrontal cortex (MPFC) (channels 5–8), Right MPFC (channels 9–12) and Right DLPFC (channels 13–16) to investigate the differences in WM activation in the prefrontal cortex.

## Statistical analysis

SPSS software (IBM Corp, 2010) was used to perform the statistical analysis. To find the effect of task and region on WM activation, we ran a repeated measure analysis of variance (ANOVA) from each region (Left and right MPFC and DLPFC) and tasks (Auditory vs. Visual) as within-subject variables. To find the effect of task on behavioral measures such a reaction time and accuracy, the repeated measure analysis was performed. We ran the multivariate ANOVA to compare the WM activation and VARK scores between HP and NP groups. To control for age in our analysis, we included age as a covariate in repeated measure and multivariate ANOVA. Multiple 2-tailed partial Pearson correlation were also conducted to find the correlation of WM activation level with, reaction time, accuracy and VARK score, while controlling for age. All the tests were performed at a significance level of < 0.05.

## Results

### Reaction time

Using within-subject test, we found the significant effect of task on reaction time (millisecond) (F(1,27) = 26.9, p<0.0005), while controlling for age, where the subjects had faster reaction time during the visual task There was no correlation between reaction time and WM activation during visual task in regions of left DLPFC (r = 0.21, p = 0.28), left MPFC (r = 0.11,p = 0.57), right MPFC (r = 0.06,p = 0.76) and right DLPFC(r = 0.019, p = 0.92). The same trend was observed during auditory task in left DLPFC (r = -0.18, p = 0.38), left MPFC (r = -0.1, p = 0.96), right MPFC (r = 0.04,p = 0.84) and right DLPFC(r = -0.14, p = 0.49) regions.

### Tasks and regions

**Fig 3:**
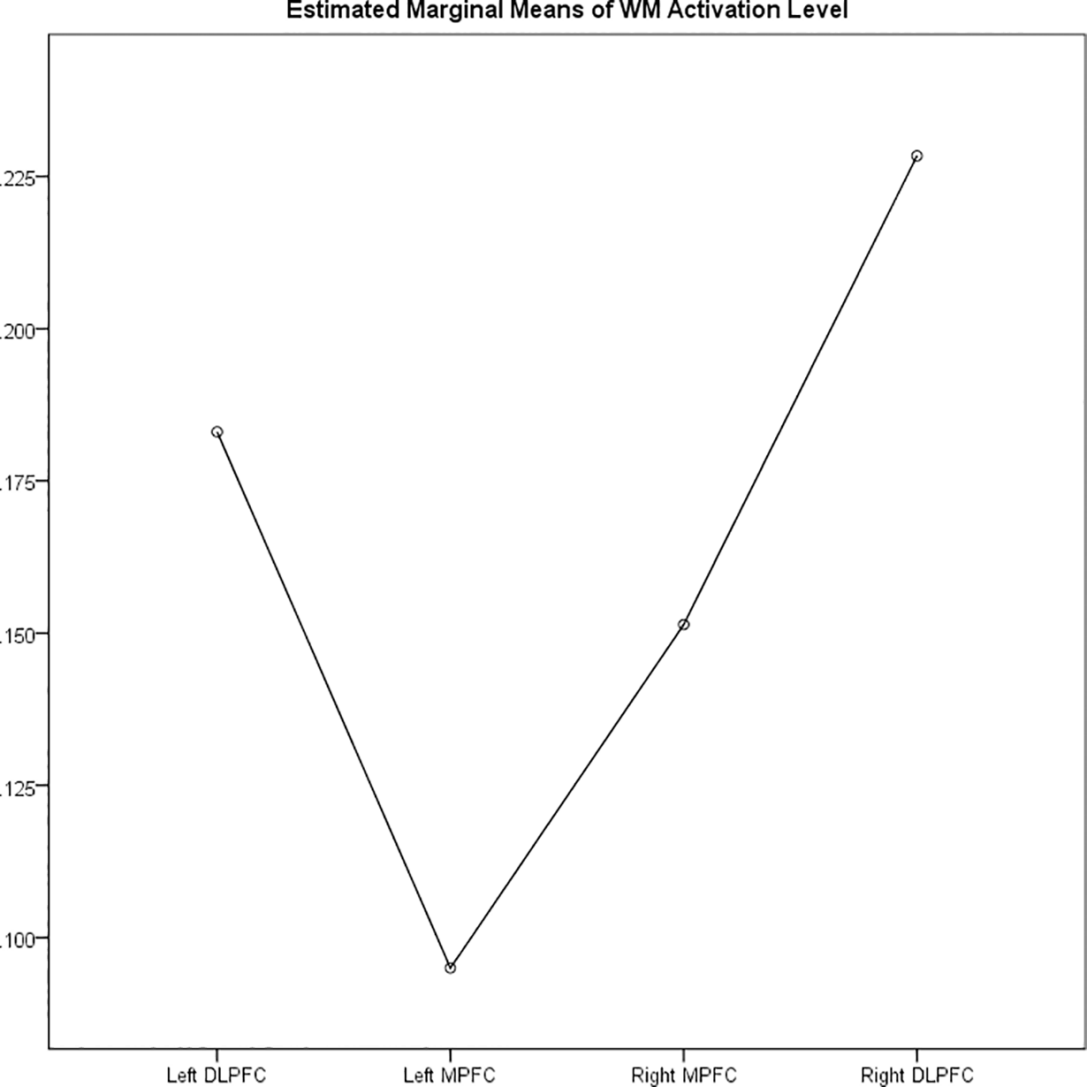
Estimated marginal means of WM activation level across all tasks in different PFC regions.

We conducted the repeated measure to examine the within-subject effects of task (visual vs. auditory) and regions (Left DLPFC, Left MPFC, Right MPFC and Right DLPFC) on the WM activation level, while controlling for age. There was no significant effect of region (F(3,47.9) = 2.05, p = 0.14), or task (F(1,23) = 1.7, p = 0.23). There was no interaction between age and region (F(2.1, 47.98) = 0.45) or age and task (F(1,23) = 0.2, p = 0.2). Based on pairwise comparison both left DLPFC and right DLPFC showed significantly higher activation compare to left MPFC (p = 0.009 and p = .012, respectively). Moreover, the right DLPFC showed significantly higher activation compared to right MPFC (p = .034) (Fig 3).

### Accuracy

**Fig 4:**
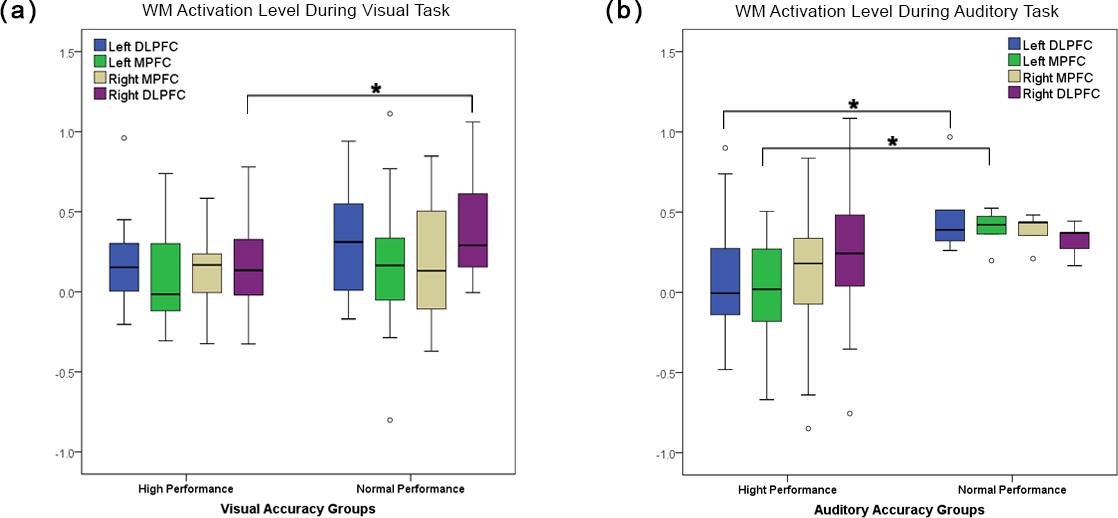
Differences in activation between high performance (HP) and normal performance (NP) groups. Both auditory and visual NP groups showed higher activation in PFC compared to their high-performance cohorts in right DLPFC during visual (a) and left DLPFC and MPFC during auditory (b) tasks.

The overall accuracy scores across all subjects were 93.6 and 92.9, for auditory and visual task, respectively. There was no significant difference between the auditory and visual accuracy scores (F(1,27) = 0.27, p = 0.67).Subjects were divided into two groups of high performance (HP) (accuracy score>90%) and normal performance (NP) based on their accuracy score during auditory and visual tasks separately (N_HP(Visual)_ = 18 and N_HP(Auditory)_ = 22). This value was selected based on the previous literature [61,62] as well as the lower bound of the 95% confidence interval of the mean accuracy from auditory 90.1 and visual 89.3 tasks. Fifteen subjects were HP in both groups. During performance of the visual task, visual HP subjects showed significantly lower WM activation compared to NP groups in right DLPFC region (F(1,26) = 6.56, p = 0.042) (Fig 4(A)). For the auditory task, HP subjects showed significantly lower activation in the left DLPFC(F(1,26) = 5.55, p = 0.027), and left MPFC F(1,26) = 5.22, p = 0.032) (Fig 4(B)). Similarly, during visual task, accuracy score showed significant negative correlation with WM activation in right DLPFC (r = -0.4, p = 0.038), while during auditory task the accuracy score showed trend of negative correlation in left DLPFC (r = -0.38, p = 0.059). Results also showed no differences in reaction time between HP and NP groups during the performance of either the auditory (F(1,26) = 0.93, p = 0.34) or visual task (F(1,26) = 1.64, p = 0.21).

### Learning preference

**Fig 5:**
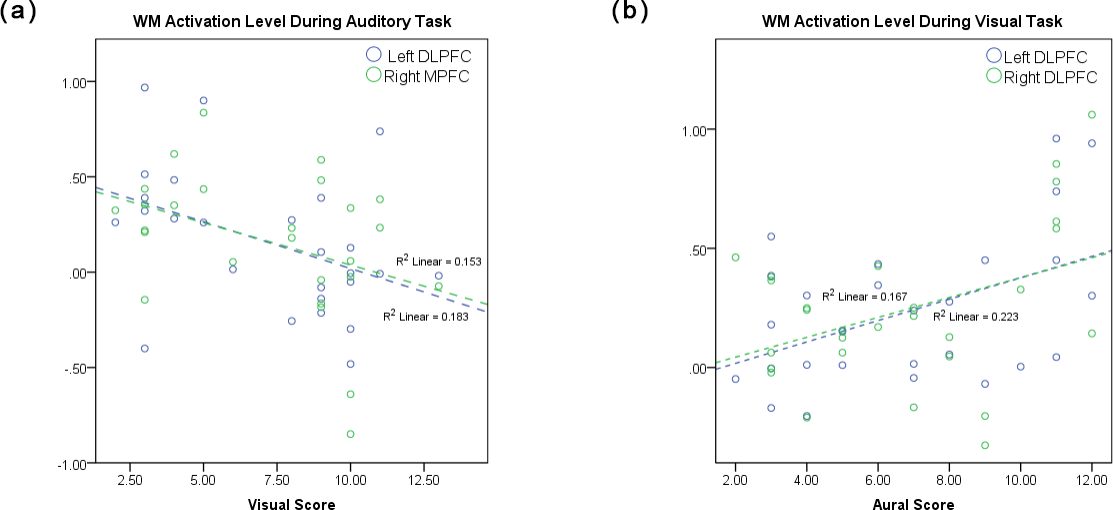
Significant correlation between VARK score and level of activation. (a) in left DLPFC VARK visual score was correlated with level of activation during performance of auditory task while (b) VARK aural score was correlated with level of activation in left and right DLPFC during performance of visual task.

We found a significant correlation between learning preference (based on VARK scores: Visual and Aural) and WM activation during both the visual and auditory tasks. While controlling for age, results showed a significant negative correlation between VARK visual score and WM activation during auditory task in left DLPFC (r = -0.43, p = 0.031) and right MPFC (r = 0.-41, p = 0.043) (Fig 5(A)). Subjects with higher VARK visual scores showed less activation during the visual task in right MPFC as well as during auditory task in left DLPFC and right MPFC compared to those with lower visual scores. The aural score showed positive correlation with the WM activation during visual task in left DLPFC (r = 0.46, p = 0.015) and right DLPFC (r = 0.39, p = 0.041) during the visual task, where subjects with higher aural score exhibited more activation compared to those with lower aural scores while processing visual stimuli (Fig 5(B)).

### Performance groups and learning preferences

**Fig 6:**
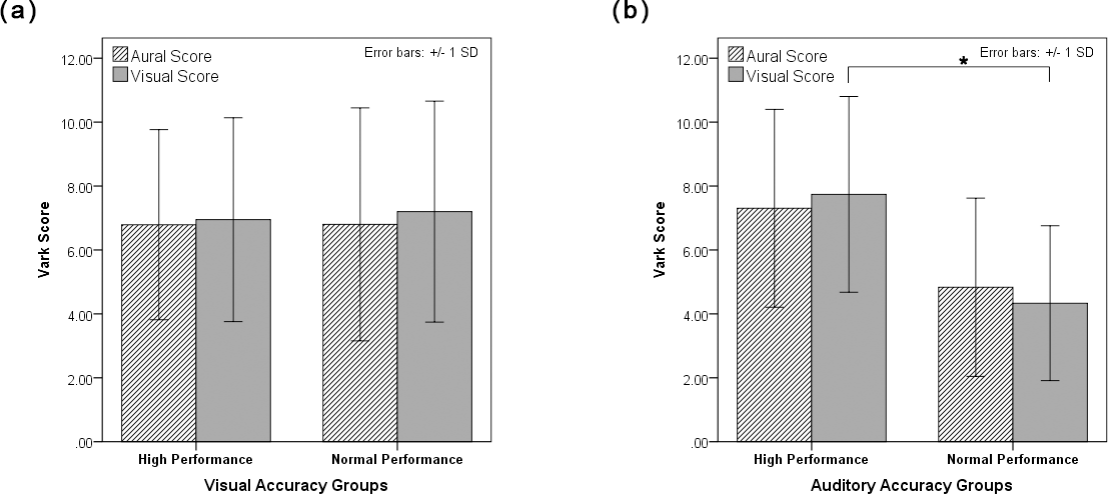
Differences between high performance and normal performance groups in visual and aural VARK scores. (a) during performance of visual task and (b) during performance of auditory task.

We found that the auditory high performance group had significantly higher visual score (F(1,26) = 6.3, p = 0.02) compared to the normal performance group but not for aural score (F(1,26) = 2.86, p = 0.1) (Fig 6(B)). We did not find any differences in VARK scores between the visual performance groups in visual scores (F(1,26) = 0.032, p = 0.86) and aural scores(F(1,26) = 0.003, p = 0.95) (Fig 6(A)). We examined the correlation between VARK scores and accuracy, while controlling for age, and we found trend of positive correlation between visual score and accuracy during auditory task. (r = 0.391, p = 0.06).

## Discussion

In this study we examined the influence performance and learning preferences during the visual and auditory N-Back working memory task, while controlling for age. The results of this study indicate that activation during the N-back task can be influenced by learning style, performance, and characteristics of the presented stimuli. The results showed differences in activation based on performance level. Specifically, normal performing group showed higher activation compared to the high performing group in the left DLPFC and MPFC regions during auditory task, while for visual tasks the activation difference was prominent in the right DLPFC Further analysis showed a correlation between learning style and activation in the DLPFC. We found a positive correlation between VARK aural score and activation during the visual n-back in the left and right DLPFC and negative correlation between visual score and activation during the auditory n-back in the left DLPFC.

Given the wide age range in our sample, we have controlled for the age variable throughout our analysis. Controlling for age ensures that the measured correlations are not influenced by this factor. Conducting partial correlation using zero-order correlation as well as using age as a controlling variable and the results from both analysis yield the similar trend, indicating that age had very small influence in relationship between other variables such as VARK score, WM activation and performance.

The role of PFC in working memory processing, attention, and executive function has been long investigated. It has been shown that PFC play roles in encoding the task relevant WM information as well as attentional control that can help with performance of WM [2,5]. For instant, in monkeys with PFC lesion, attending to the stimuli was disrupted. Therefore, it has been suggested that DLPFC helps with information maintenance [63] and to focus attention to the represented stimulus and control of cognitive processing by means of executive function [64]. Several studies have focused on the importance of the presented stimuli during working memory tasks. It has been shown that PFC activity during verbal (letter) working memory tends to be left lateralized [16]. Schumacher et al. [20] showed that there was higher activation during auditory WM compared to visual WM in Broca’s area. Crottaz-Herbette et al. [17] used fMRI to investigate the difference in activation between tasks that presented auditory and visual stimuli and found increased activation in the left dorsolateral prefrontal cortex when subjects were presented with an auditory stimulus. Crottaz-Herbette et al. concluded that modality-specific increases in left hemisphere activation were due to a bias for verbal information processing as part of the encoding, storage, and manipulation of verbal working memory. Studies comparing visual vs. auditory n-back task have shown the bilateral activation in DLPFC regions, where left DLPFC exhibited greater activation during auditory task compared to the visual task exhibiting higher activation in posterior brain regions [19]. Although we did not find the effect of side, the pairwise comparison revealed higher activation in left and right DLPFC compared to left and right MPFC, respectively We hypothesize that this finding could be due to an influence of factors such as learning style and accuracy as well as involvement of different underlying neural networks other than the PFC as it will be discussed further in this section.

Few studies have examined the effect of task performance during working memory tasks on brain activation. Ogawa et. al. [18] showed that during visuospatial working memory tasks, subjects with better performance had higher activation in PFC regions. Another fNIRS study, showed that high performance groups showed an increase in activation in the DLPFC from 0-back to 2-back, whereas the normal group showed lower activation in the 2-back compared to the 0-back [62]. Study of task performance in elderly population have shown that the high-performance groups exhibited higher increase in activation in right PFC during performance of spatial WM task [65]. Those elderlies who were considered low performer revealed load related performance decline and showed larger load related increase in bilateral prefrontal cortex. Authors further deduced that such observation could be related to age group limitation of the study. Another study has shown the effect of subject performance on activation of prefrontal cortex during WM has shown increase in activation in low performing groups in DLPFC compared to high-performing subjects [66]. Based on our results, differences in level of activation between HP and NP groups were specific to both task and region. During auditory task, HP individuals showed decreased activation compared to NP individuals in left DLPFC region. The observed differences in left PFC region during the auditory task is in line with other studies that have shown activation in response to auditory WM task and auditory stimuli in region of left PFC [17,67], which is known to be related to auditory processing [19]. During the visual task the HP group exhibited less WM activation in comparison with NP group, prominently in the right DLPFC, an area which has been shown to be related to visual processing of shapes [68] as well as visuospatial information [69]. This implies that during the visual task there might be a difference in how HP and NP individuals might use image-based encoding of the letters that were presented during the visual task. In shadow of neural efficiency hypothesis where task performance can be related to the cognitive abilities and neural efficiency [70], the lower activation in HP groups also suggests that while performing the given task, compared to NP cohorts, the HP group might be more efficient (exhibiting less activation) in the corresponding PFC regions. In the future, administration of IQ test can clarify the role of neural efficiency, since there has been evidences of interaction between IQ and brain activation and performance [71–73].

The observed differences in activation between HP and NP groups can also be influenced by different learning strategies that each group uses. We first explored the correlation of learning style and WM activation and further examined the differences in the learning preferences between HP and NP groups. Learning preference can affect neural activation during working memory tasks. Behavioral research has shown the differences in WM strategies based on the learning preferences of the subject [21] as well as the interaction between working memory capacities and cognitive style [23]. For instance, those with image preferences (imagers) are more likely to better memorize if the pictorial presentation is used instead of the verbal. Study by Kraemer et.al [22] has shown that subjects with visual style tend to convert linguistic information into a visual mental image that can increase the activation in fusiform regions. The correlation between activation in phonological brain region and verbal cognitive style was also found but only when subjects with verbal preferences were presented with images, indicating the conversion of pictorial information to linguistic representation. Similarly, the correlation of visual and auditory scores with WM activation in this study confirms the possible association between learning style and task nature. Our results show that subjects with higher VARK aural scores exhibited less efficient neural activation (higher activation) during the visual task but not the auditory task, indicating that those with higher auditory preferences work harder while processing the visual stimuli. This increase was noticeable in bilateral DLPFC, the region contributing to WM processing and maintenance as well as encoding the visual representation to that of auditory. This elucidates the possible effect of visual to auditory conversion process in these subjects, which can also be more intensive in terms of maintenance and activation. Subject with higher VARK visual score on the other hand showed lower activation during the auditory task in left DLPFC. The negative correlation between visual score and WM activation in left DLPFC during auditory stimuli shows the possibility of such compensatory strategies. The visual subjects perhaps tend to use visual encoding of the auditory stimuli instead of auditory encoding, which can be manifested as an increase in visual cortex. Processing auditory information as a pictorial format in visual and fusiform regions instead of auditory regions in more visual subjects can be manifested as lower activation in auditory regions.

Further analysis also showed that during performance of the auditory task, HP groups have significantly higher visual VARK scores compared to NP groups. This result can explain the earlier findings of lower WM activation in HP groups in relation with two assumptions. First, the result would suggest that observed decrease in WM activation of the HP groups compared to NP participants during auditory task in left PFC could be owing to the learning preference of the HP group. Auditory HP groups seem to have higher visual scores, which has a negative correlation with the WM activation during the auditory task in left DLPFC. This in turn can lead to reduced WM activation in HP groups in left PFC regions as observed in section 3.4. Secondly, these findings may relate to the neural efficiency hypothesis of intelligence, which shows a negative correlation between cognitive abilities and neural activation. Specifically, individuals with more ability in each type of processing might show more efficient neural activity indicated by decrease in measures of activation in task specific regions. Neubauer et al. [74] showed that while this pattern is not always present, it does hold for frontal activation when subjects are given easy to moderately complex tasks. Therefore, understating of interconnection between learning preference and performance can become an important aspect of underlying neural activation in presence of the cognitive task.

This study demonstrates the effect of learning style and task performance on PFC activation during an n-back task. We showed evidence that lower activation in the auditory HP group was related to their learning strategy and that activation in PFC can be affected by preferred learning strategies. Therefore, we suggest the consideration of the impact of other measures, such as task performance and learning style, on the interpretation of working memory results.

### Limitations

The NIRS sensor used in this study has been designed for placement on the forehead to minimize changes in signal due to hair presence. Therefore, any observed differences are limited and cannot include more extensive neural regions known to be involved in working memory tasks or processing of the visual information such as occipital cortex. Moreover, NIRS suffers from depth resolution, where it can only monitor the cortical activation.

Future studies on the effects of learning style on neural activation should measure IQ (i.e. Verbal and Non-verbal) in addition to learning preferences to clarify the relationship between age, activation, and verbal and nonverbal ability [75], as well as the possible effect of cognitive and learning style, especially in context of neural efficiency theory. In future we will increase the number of trials to reduce the possible variabilities in activation due to rate of learning in each subject. Increasing the number of subjects and comparing the age groups can further elucidate the variations in WM activation on basis of developmental changes.

## Conclusion

Using fNIRS, we explored the effect of several factors such as age, learning style, and performance on both auditory and visual N-back tasks on brain activation in the PFC. These preliminary results suggest an important role for task variables and individual differences on neural activation. The result of this study highlights the importance of individual differences and their cognitive capacities and preference in performance of functional tasks such as working memory. These findings can have implications toward understanding patient populations with auditory or visual processing difficulties (e.g. dyslexia, hearing impairment), including a potential role for emphasis on learning style to improve WM and underlying cognitive processing.
